# Increased impulsivity and higher odds of compulsive shopping among cabergoline-treated patients with prolactinoma: a case-control study

**DOI:** 10.1007/s11102-026-01728-z

**Published:** 2026-07-24

**Authors:** Karla Borges Daniel, Andrea Glezer, Nara Lima de Queiroz, Silvia Regina Correa-Silva, Marília Bortolotto Felippe Trentin, Mariana Tazima Fujiwara, Cristina Laguna Benetti-Pinto, Leandro Kasuki, Leonardo Andrade Gontijo Tavares, Pedro Weslley Souza do Rosário, Heraldo Mendes Garmes

**Affiliations:** 1 Division of Endocrinology, Department of Clinical Medicine, Faculty of Medical Sciences, State University of Campinas (Unicamp), Campinas, Brazil; 2 Neuroendocrine Unit, Division of Endocrinology and Metabolism, Hospital das Clínicas, University of São Paulo Medical School, São Paulo, Brazil; 3 Neuroendocrinology Unit, Division of Endocrinology and Metabolism, Paulista School of Medicine, Federal University of São Paulo (EPM-Unifesp), São Paulo, SP Brazil; 4 Department of Obstetrics and Gynecology, School of Medical Sciences, State University of Campinas (UNICAMP), Campinas, SP Brazil; 5 Neuroendocrinology Research Center/Endocrinology Division, Medical School and Hospital Universitário Clementino Fraga Filho, Universidade Federal do Rio de Janeiro, Rio de Janeiro, Brazil; 6 Neuroendocrinology Service, Santa Casa de Misericórdia de Belo Horizonte, Belo Horizonte, MG Brazil; 7 Rua Vital Brasil, 80, Cidade Universitária UNICAMP, Campinas, SP 13083-888 Brazil

**Keywords:** Prolactinoma, Cabergoline, Impulsivity, Impulse control disorders

## Abstract

**Abstract:**

Dopamine agonists, particularly cabergoline, are the first-line treatment for prolactinomas, but have been associated with impulse control disorders (ICDs). However, data on impulsivity and related behaviors in this population remain limited and inconsistent.

**Objective:**

To evaluate impulsivity and ICDs in patients with prolactinoma treated with cabergoline and to compare these findings with healthy controls.

**Methods:**

This case–control study included 131 patients with prolactinoma receiving cabergoline and 131 healthy controls. Impulsivity was assessed using the Barratt Impulsiveness Scale (BIS-11), and ICDs were evaluated using specific questionnaires addressing hypersexuality, gambling, compulsive shopping, and punding. Multivariable analyses were performed to assess independent associations.

**Results:**

Patients treated with cabergoline exhibited higher overall impulsivity, reflected by increased BIS-11 scores and a higher proportion of individuals with increased impulsivity (BIS-11 ≥ 60). Attentional impulsivity remained significantly higher in patients after multivariable adjustment. Patients also had more than fourfold higher odds of compulsive shopping compared with controls, whereas no significant differences were observed for other ICDs. Lower educational level was also associated with higher impulsivity across all BIS-11 domains and with compulsive shopping.

**Conclusion:**

Patients with prolactinoma treated with cabergoline exhibit increased impulsivity, particularly in the attentional domain, along with higher odds of compulsive shopping. These results highlight the role of dopaminergic modulation and the influence of sociodemographic factors, supporting the importance of actively assessing impulsivity during clinical follow-up.

**Supplementary Information:**

The online version contains supplementary material available at https://doi.org/10.1007/s11102-026-01728-z.

## Introduction

Dopamine agonists (DAs) are the first-line therapy for prolactinomas, which account for approximately 50% of all pituitary adenomas. The primary goals of treatment are to reverse hypogonadotropic hypogonadism and reduce tumor volume [1–3]. Cabergoline is preferred due to its longer half-life, greater efficacy, and favorable tolerability profile [2, 4]. Although generally well tolerated, common adverse effects are nausea, dizziness, headache, and postural hypotension, affecting up to 29% of patients treated with cabergoline [4, 5]. In addition to these well-recognized adverse effects, increasing attention has been directed toward potential neuropsychiatric effects of DAs [2].

The first report linking cabergoline to pathological gambling and paranoid delusions in a patient treated for prolactinoma was published in 2007 [6]. At that time, DAs used in Parkinson’s disease were already associated with an increased risk of developing impulse control disorders (ICDs), defined as the inability to resist an impulse or urge to engage in behavior despite harmful consequences [7]. Since then, several studies have investigated ICDs in patients treated with DAs for hyperprolactinemia, reporting a wide range of frequency rates. However, most studies are observational, have relatively small sample sizes, lack a standardized control group, and in some cases also include patients with acromegaly [8, 9]. In addition, the methods used to screen for ICDs are heterogeneous [8, 9]. Consequently, the true prevalence and characteristics of impulsivity in patients with prolactinoma treated with DAs remain uncertain.

Therefore, this study aimed to evaluate impulsivity and impulse-related behaviors in patients with prolactinoma receiving cabergoline using the Barratt Impulsiveness Scale (BIS-11) and questionnaires assessing sexual, shopping, and gambling behaviors, and to compare these findings with those of healthy controls in a case–control study.

## Materials and methods

### Study design and participants

This was a case-control study including patients with prolactinoma treated with cabergoline (*n* = 131) and healthy controls (*n* = 131). The study was approved by the Research Ethics Committee of the University of Campinas (UNICAMP), Brazil (CAAE: 46682520.5.0000.5404). Written informed consent was obtained from all participants.

Inclusion criteria were patients aged ≥ 18 years with a diagnosis of prolactinoma who had been treated with cabergoline for at least 3 months. Exclusion criteria included non-tumoral hyperprolactinemia, hyperprolactinemia associated with stalk effect, patients with prolactinoma who had discontinued cabergoline, acromegaly, and psychiatric disorders involving psychosis or the use of antipsychotic drugs.

Participants were recruited between January 2021 and December 2025. A total of 26 patients with prolactinoma receiving cabergoline were recruited during follow-up visits at the Endocrinology outpatient clinic of the Hospital de Clínicas, UNICAMP, and completed the questionnaires in person during these visits.

The remaining 105 patients completed the same questionnaires through online forms. These participants were either patients receiving follow-up care who were unable to complete the questionnaires in person during their visit, or patients under follow-up with experienced neuroendocrinologists who identified eligible participants based on their medical records. The diagnosis of prolactinoma was established based on clinical, biochemical, and imaging findings according to current clinical guidelines [2, 10]. Online recruitment was conducted in accordance with approval from the institutional ethics committee. The same questionnaires and instructions were used for both in-person and online participants to ensure consistency in data collection.

The control group consisted of healthy volunteers who completed the questionnaires online and were recruited at the same hospital, among staff members, companions of patients attending outpatient clinics, and individuals from the hospital community. Controls had no history of pituitary disease, psychotic disorders, or antipsychotic use, and were recruited to obtain a group with sociodemographic characteristics similar to the patient group.

Educational level was assessed in all participants according to the highest completed level of formal education and categorized as up to high school education versus complete or incomplete college degree.

History of anxiety or depression and psychiatric medication use were assessed by self-report using yes/no questions.

E-mail addresses were used as unique identifiers in the online survey, and duplicate responses were identified and excluded from the analysis.

### Assessment of impulsivity and impulse control disorders

#### The Barratt Impulsivity Scale (BIS-11)

The Barratt Impulsiveness Scale (BIS-11) is a self-report questionnaire consisting of 30 items designed to assess impulsivity. It is subdivided into three components: motor impulsivity (acting without thinking), attentional impulsivity (rapid decision-making and difficulty maintaining focus), and non-planning impulsivity (lack of future orientation). Each item is rated on a four-point Likert scale (1 = rarely/never, 2 = occasionally, 3 = often, and 4 = almost always), resulting in a total score ranging from 30 to 120, with higher scores indicating greater impulsivity. The questionnaire has been validated in the Brazilian Portuguese language [11]. Although no established cut-off exists for a positive screening of impulsivity, a previous study suggested a threshold of ≥ 60 for screening purposes [12].

#### ICD-specific questionnaires

Screening for hypersexuality, gambling, compulsive shopping, and punding was performed using a Portuguese-adapted four-part questionnaire previously used by Bancos et al. [13]. The questionnaire was adapted for use in the present study without modification of its core structure.

Hypersexuality was defined as answering “yes” to increased sexual drive and having a total score ≥ 2. Compulsive shopping was defined as a total score ≥ 9. Gambling behavior was defined as reporting participation in any type of game of chance and having a total score ≥ 5. Punding was defined as a total score ≥ 4.

#### Sample size calculation

The sample size was calculated for the comparison of BIS-11 scores between two independent groups. Assuming a standard deviation of 11 points based on previous literature [12], a minimum clinically relevant difference of 5 points, a two-sided alpha level of 0.05, and 90% statistical power, the estimated sample size was 102 participants per group. The final sample included 131 participants in each group, exceeding this estimate.

### Statistical analysis

Quantitative variables were described as median (range), and qualitative variables as absolute frequencies and valid percentages (%), excluding missing data from the denominator. The distribution of quantitative variables was assessed using the Shapiro–Wilk test. As quantitative variables showed a non-normal distribution, non-parametric methods were applied.

Comparisons between groups were performed using the Mann–Whitney U test for quantitative variables. Associations between categorical variables were assessed using the Pearson chi-square test or Fisher’s exact test, as appropriate.

To evaluate the independent association between clinical group and impulsivity outcomes, multivariable models were constructed. For continuous outcomes (BIS-11), generalized linear models (GLMs) were used. Different distribution families (Gaussian and Gamma) and link functions (identity and log) were tested, and the final model was selected based on the lowest Akaike information criterion (AIC).

For the categorical outcome of increased impulsivity (BIS-11 ≥ 60), logistic regression models were applied, with results expressed as odds ratios (ORs) and 95% confidence intervals (CIs).

Both univariable and multivariable analyses were performed. Multivariable models were adjusted for predefined clinically relevant confounders, including age, sex, and educational level. An interaction term between group and educational level was included to assess potential effect modification.

Estimated marginal means (predicted values) were calculated from the final multivariable models to estimate the joint effects of clinical group and educational level on impulsivity outcomes. These estimates were back-transformed to the original scale when applicable.

A two-sided p value < 0.05 was considered statistically significant. All analyses were performed using R statistical software (version 4.3.1).

## Results

### Participant characteristics

A total of 262 participants were included in the study: 131 patients with prolactinoma treated with cabergoline and 131 healthy controls. Baseline demographic and clinical characteristics of the participants are presented in Table 1.

**Table 1:** Demographic and clinical characteristics of the participants

	Prolactinoma group (*n* = 131)	Control group (*n* = 131)	*p*-value
Age, years	41 (22–72)	38 (18–70)	0.412
Female sex, n (%)	89 (67.9)	89 (67.9)	1
Education level, n (%)			
High school or less	78 (59.5)	63 (48.1)	0.083
College degree	53 (40.5)	68 (51.9)	
Current smoking, n (%)	6 (4.6)	9 (6.9)	0.595
Alcohol intake, n (%)	12 (9.2)	36 (27.5)	**< 0.001**
Anxiety or depression, n (%)	23 (17.6)	23 (17.6)	1
Antidepressants use, n (%)	16 (12.2)	15 (11.5)	1
Family psychiatric illness, n (%)	48 (36.6)	37 (28.2)	0.187

Continuous variables are presented as median (range), and categorical variables as number (percentage)

The two groups were similar regarding age and sex. The median age was 41 years in the prolactinoma group and 38 years in the control group (*p* = 0.412), and women accounted for 67.9% of participants in both groups.

Educational level was also similar between groups (*p* = 0.083). Alcohol consumption was more frequently reported in the control group (27.5% vs. 9.2%, *p* < 0.001), whereas no significant differences were observed in smoking status, anxiety or depression, family history of psychiatric illness, or antidepressant use. The classes of antidepressants were similar in both groups and consisted of selective serotonin reuptake inhibitors (SSRIs) and tricyclic antidepressants.

The median duration of cabergoline use was 8 years (3 months–30 years), and the median dose was 0.5 mg (0.25–3.5 mg). 17 patients (13.0%) had undergone transsphenoidal surgery, and 6 (4.6%) had received radiotherapy. Hormone replacement therapy, including sex steroids, levothyroxine, and glucocorticoids, was used in 14 (10.7%), 22 (16.8%), and 4 (3.1%) patients, respectively. In the control group, 8.4% were receiving levothyroxine for primary hypothyroidism, with no significant difference compared with the prolactinoma group (*p* = 0.626).

### BIS-11 and increased impulsivity

The median BIS-11 score was higher in patients with prolactinoma receiving cabergoline compared with controls (61 vs. 56, *p* = 0.039) (Supplementary Figure S1), as was the proportion of participants with scores ≥ 60 (*p* = 0.002). Subscale analysis of the BIS-11 showed that motor and attentional impulsivity were similar between groups (*p* = 0.373 and *p* = 0.090, respectively), whereas non-planning impulsivity was higher in patients with prolactinoma (*p* = 0.022). However, these findings were not all maintained after multivariable adjustment. Detailed BIS-11 results are presented in Table 2.

**Table 2:** BIS-11 and impulse control disorder

	Prolactinoma group (*n* = 131)	Control group (*n* = 131)	*p*-value
BIS-11			
Total score	61 (35–92)	56 (39–90)	**0.039**
Motor	19 (11–31)	18 (11–38)	0.373
Attentional	16 (8–27)	15 (8–30)	0.090
Non-planning	26 (15–39)	24 (14–36)	**0.022**
BIS-11 ≥ 60, n (%)	78 (59.5)	52 (39.7)	**0.002**
Impulse control disorders			
Hypersexuality, n (%)	8 (6.1)	3 (2.3)	0.218
Compulsive shopping, n (%)	18 (13.7)	18 (13.7)	1
Gambling, n (%)	5 (3.8)	4 (3.1)	1
Punding, n (%)	8 (6.1)	10 (7.6)	0.807

Continuous variables are presented as median (range), and categorical variables as number (percentage)

In multivariable analysis (Table 3), belonging to the cabergoline-treated prolactinoma group remained independently associated with an 8% increase in BIS-11 (exp[β] = 1.08, 95% CI 1.01–1.15, *p* = 0.015). Lower educational level was also independently associated with a 16% increase in BIS-11 (exp[β] = 1.16, 95% CI 1.09–1.23, *p* < 0.001), whereas no significant associations were observed for age (*p* = 0.200) or sex (*p* = 0.100). Furthermore, no significant interaction between clinical groups (cabergoline-treated prolactinoma vs. controls) and educational level was observed (*p* = 0.083), indicating that the effect of educational level was similar across groups, while patients remained more impulsive overall.

**Table 3:** Multivariable generalized linear models for BIS-11 total and subdomain scores

Outcome	Variable	exp(β)	95% CI	*p*-value
BIS-11 total score	Prolactinoma group treated with cabergoline	1.08	1.01–1.15	**0.015**
	Age	1.00	1.00–1.00	0.200
	Male sex	0.96	0.92–1.01	0.100
	High school education or less	1.16	1.09–1.23	< 0.001
	Group × educational level	0.93	0.86–1.01	0.083
Motor impulsivity	Prolactinoma group treated with cabergoline	1.08	0.99–1.16	0.070
	Age	1.00	1.00–1.00	0.400
	Male sex	0.98	0.92–1.04	0.400
	High school education or less	1.15	1.07–1.24	< 0.001
	Group × educational level	0.90	0.81–1.01	0.066
Non-planning impulsivity	Prolactinoma group treated with cabergoline	1.06	0.99–1.14	0.087
	Age	1.00	1.00–1.00	0.600
	Male sex	0.96	0.91–1.01	0.082
	High school education or less	1.18	1.11–1.26	< 0.001
	Group × educational level	0.96	0.88–1.05	0.400
Attentional impulsivity	Prolactinoma group treated with cabergoline	1.11	1.01–1.22	**0.025**
	Age	1.00	1.00–1.00	0.078
	Male sex	0.94	0.88–1.01	0.089
	High school education or less	1.14	1.05–1.25	0.003
	Group × educational level	0.90	0.80–1.02	0.110

Models were adjusted for age, sex, and educational level. Estimates are presented as exponentiated beta coefficients, representing ratios of means. Generalized linear models with log link were used. Reference categories were control group, female sex, and college degree

Estimated marginal means derived from the multivariable model were examined to aid interpretation of the non-significant interaction between group and educational level. Among individuals with a college degree, patients had higher predicted BIS-11 scores compared with controls (58.74 vs. 54.45). Among individuals with lower educational attainment, predicted BIS-11 scores were also slightly higher in patients than in controls (63.33 vs. 63.17). These findings are consistent with the absence of a statistically significant interaction between group and educational level (*p* = 0.083).

Similarly, patients had higher odds of increased impulsivity (BIS-11 ≥ 60) than controls (OR = 2.58, 95% CI 1.20–5.70, *p* = 0.015). Lower educational level was also associated with this outcome (OR = 3.86, 95% CI 1.85–8.35, *p* < 0.001). No significant interaction between group and educational level was observed (*p* = 0.50).

In subscale analyses, after adjustment for potential confounders (age, sex, and educational level), the association between prolactinoma group and BIS-11 subscales remained significant only for attentional impulsivity, corresponding to an 11% increase compared with controls (exp[β] = 1.11, 95% CI 1.01–1.22, *p* = 0.025), whereas no significant associations were observed for motor or non-planning impulsivity. Lower educational level was associated with higher scores across all BIS-11 subdomains in both patients and controls, including motor impulsivity (*p* < 0.001), non-planning impulsivity (*p* < 0.001), and attentional impulsivity (*p* = 0.003). The complete results of all multivariable models, including interaction terms, are provided in Supplementary Tables S1–S5.

### Specific impulse control disorders

In univariable analysis, no significant differences were observed in the prevalence of hypersexuality (*p* = 0.218), compulsive shopping (*p* = 1.000), gambling (*p* = 1.000), or punding (*p* = 0.437) (Table 2).

However, in multivariable analysis, prolactinoma patients receiving cabergoline had higher odds of compulsive shopping compared with controls (OR = 4.47, 95% CI 1.33–17.8, *p* = 0.015). Compulsive shopping was more prevalent in women, as male sex was associated with substantially lower odds (OR = 0.11, 95% CI 0.02–0.38, *p* < 0.001). Lower educational level was also associated with higher odds (OR = 4.52, 95% CI 1.46–17.2, *p* = 0.008). A significant interaction between group and educational level was observed (*p* = 0.002), indicating that the association between patient group and compulsive shopping varied according to educational level.

In contrast to compulsive shopping, no significant differences in the prevalence of punding, hypersexuality, or gambling were observed between groups in multivariable analyses. Age was inversely associated with punding (OR = 0.93, 95% CI 0.87–0.97, *p* = 0.002), whereas male sex was associated with hypersexuality (OR = 4.00, 95% CI 1.15–15.9, *p* = 0.029) and gambling (OR = 5.90, 95% CI 1.44–30.1, *p* = 0.013). Educational level was not significantly associated with these ICDs. Complete results of the multivariable analyses for ICD outcomes are provided in Supplementary Tables S6–S10.

### Additional associations

No significant association was observed between depression or anxiety and BIS-11 ≥ 60 (*p* = 0.129), hypersexuality (*p* = 1.000), or gambling (*p* = 1.000); however, significant associations were observed with compulsive shopping and punding (*p* < 0.001) in the overall sample.

### Cabergoline dose and treatment duration

The median cabergoline dose was higher in patients with BIS-11 ≥ 60 compared to those with lower scores (1.0 vs. 0.5 mg, *p* = 0.021). No differences in dose were observed according to the presence of hypersexuality, gambling, or compulsive shopping.

Although the median cabergoline dose was similar (0.5 mg) between patients with and without punding, the distribution differed significantly, with higher dose values observed among patients without punding (0.25–3.5 vs. 0.25–1.5 mg; *p* = 0.033).

Treatment duration was not associated with BIS-11 scores or specific ICDs.

## Discussion

This study found that prolactinoma patients receiving cabergoline exhibited higher overall impulsivity, as reflected by increased BIS-11 scores and a higher proportion of individuals with increased impulsivity (BIS-11 ≥ 60). After multivariable analysis, attentional impulsivity, characterized by impaired sustained attention, remained significantly higher in patients treated with cabergoline than in controls. Patients also had more than fourfold higher odds of compulsive shopping compared with controls. Conversely, no significant differences were observed in the prevalence of other ICDs between groups. Notably, lower educational level emerged as a consistent factor associated with higher impulsivity across all BIS-11 domains, independent of clinical group, and was also associated with higher odds of compulsive shopping.

The mechanisms potentially underlying our findings may involve dopaminergic modulation of mesocorticolimbic pathways. D2 and D3 receptors, which are expressed in fronto-striatal and limbic circuits involved in reward processing, motivation, and cognitive control, play a key role in the regulation of impulsivity [14, 15]. Cabergoline is a dopamine agonist with high affinity for D2 and substantial affinity for D3 receptors, which may explain its association with increased impulsive behaviors [16].

Our findings of higher BIS-11 scores in patients receiving cabergoline for prolactinoma, compared with healthy controls, expand on previous studies, which have reported inconsistent results regarding BIS-11 scores. A cross-sectional study including 10 hyperprolactinemic patients on DAs, 10 individuals with untreated hyperprolactinemia and 10 patients with normoprolactinemic pituitary tumors identified no differences in total, motor and non-planning scores. Nevertheless, hyperprolactinemic DA-treated patients had a higher mean attentional subscale score (16.2 ± 2.7) compared with hyperprolactinemic patients without treatment (12.3 ± 2.5) and the normoprolactinemic group (14.7 ± 4.4) (*p* = 0.04) [17]. This finding is consistent with our results and suggests that attentional impulsivity may be particularly sensitive to dopaminergic modulation, supporting the dopamine overdose hypothesis previously described in patients with Parkinson’s disease, in which dopaminergic stimulation may differentially affect neural circuits involved in attention and impulse control [18]. However, whether this model fully applies to prolactinoma patients treated with cabergoline, who do not have underlying nigrostriatal dopamine deficiency but rather dysfunction involving the tuberoinfundibular pathway, remains to be determined.

The clinical relevance of the BIS-11 was also demonstrated in a large multicenter cross-sectional study including 308 prolactinoma patients receiving DAs, in which significantly higher BIS-11 total and subdomain scores were observed among individuals with ICDs, including compulsive shopping, compulsive eating, hypersexuality, and gambling, compared with those without ICDs (*p* < 0.001). In that study, a BIS-11 total score ≥ 61 was identified as the optimal cutoff for discriminating patients with ICDs, with high sensitivity and specificity. Together with our findings, these results support the potential utility of the BIS-11 as a practical screening tool for identifying patients at increased risk of ICDs during dopamine agonist treatment [19].

In contrast, a Chinese study including treated and untreated patients with hyperprolactinemia found no significant differences in BIS-11 scores among DA-treated individuals. However, most patients were treated with bromocriptine, which may limit direct comparability with the present study [20]. Another study including a heterogeneous cohort of DA-treated pituitary adenomas likewise found no significant differences in total BIS-11 scores or subdomains compared with DA-naïve patients [12]. Celik et al. found no significant changes in BIS-11 scores during the first year of cabergoline treatment and no differences compared with patients with non-functioning pituitary adenomas and healthy controls [21]. These discrepant findings compared with ours may reflect methodological differences across studies, particularly smaller sample sizes and heterogeneous populations, which may have limited the ability to detect differences in BIS-11 scores. On the other hand, our study included a larger and better-characterized sample, as well as a healthy control group with comparable characteristics, which may have improved the detection of differences in BIS-11.

With respect to specific ICDs, no significant differences in overall prevalence were observed between patients and controls in univariable analyses. However, in multivariable analysis, patients with prolactinoma receiving cabergoline had more than fourfold higher odds of compulsive shopping compared with controls. Although the confidence interval was wide, likely due to the low prevalence of this outcome, similar associations have been reported in previous studies. A multicenter cross-sectional study demonstrated higher rates of compulsive buying among DA-treated hyperprolactinemic patients compared with controls (15.9% vs. 6.1%) [22]. Similarly, a survey using the Questionnaire for Impulsive-Compulsive Disorders in Parkinson’s Disease–Rating Scale (QUIP-RS) reported higher rates of compulsive shopping among DA–treated prolactinoma patients compared with individuals with NFPA, but without a sex-specific association [23]. In contrast, other studies have not identified differences in compulsive shopping frequency. A study using the four-part questionnaire did not find differences in compulsive shopping frequency between 77 prolactinoma patients and a control group composed of 70 patients with NFPAs (*p* = 0.14). However, treatment exposure was heterogeneous, with 29%, 53% and 18% of patients receiving bromocriptine, cabergoline, or no DA therapy at the time of the survey, respectively, which may have influenced the results [13]. In our study, compulsive shopping was more prevalent in women, as male sex was associated with substantially lower odds. This observation aligns with epidemiological data from the general population, where compulsive buying behavior is more frequently reported among women, although results remain heterogeneous across populations [24].

These divergent findings regarding specific ICDs may reflect differences in study populations and methodological approaches across studies. Most available instruments were originally developed and validated in Parkinson’s disease and later extrapolated to prolactinoma populations, which may compromise the accuracy and comparability of ICD estimates [9, 20, 22]. Furthermore, genetic polymorphisms involving dopamine pathways have been associated with ICDs in cabergoline-treated prolactinoma patients, suggesting an additional biological basis for interindividual variability in ICD susceptibility [25].

An important finding of our study was the consistent association between lower educational level and higher impulsivity across all BIS-11 subdomains, as well as compulsive shopping. Individuals with education up to high school had 3.86-fold higher odds of increased impulsivity (BIS-11 ≥ 60) compared with those holding a college degree across both groups. Estimated marginal means also suggested that the association between clinical group and impulsivity varied according to educational level. Although prolactinoma patients receiving cabergoline exhibited higher overall impulsivity scores than controls, group differences were more evident among individuals with higher educational attainment. In contrast, among individuals with lower educational levels, impulsivity scores were elevated in both groups. Nevertheless, despite the influence of educational level, cabergoline-treated patients consistently exhibited higher overall impulsivity scores than controls, suggesting that both lower educational level and cabergoline-treated prolactinoma are independently associated with increased impulsivity. Lower educational attainment is well recognized as a risk factor for impulsivity and psychiatric disorders in the general population [26, 27]. A similar pattern has been described for specific impulsive behaviors, including gambling, with a higher risk among individuals with lower educational levels, as well as compulsive shopping [28, 29]. One possible mechanism linking lower education to impulsivity is that socioeconomic deprivation impairs executive function development, particularly inhibitory control and working memory [30]. This pattern supports the idea that educational level may act as a vulnerability factor, alongside the effects of dopaminergic treatment.

Another aspect explored in our study was the relationship between cabergoline dose and impulsivity. We found that a higher median cabergoline dose was associated with BIS-11 ≥ 60; however, the magnitude of the difference was small, and there was overlap in dose ranges between patients with BIS-11 ≥ 60 and < 60. Previous studies have reported inconsistent results regarding the relationship between cabergoline dose and BIS-11 scores. Barake et al. found no correlation between cabergoline dose and BIS-11 [17], whereas Hinojosa-Amaya et al. reported an inverse correlation between total cumulative DA dose and BIS-11 score [12]. Overall, these findings suggest a weak and inconsistent relationship between DA dose and impulsivity, particularly at the relatively low doses used in prolactinoma.

Nevertheless, in our study, cabergoline dose was not associated with specific ICDs, except for punding. Although median doses were similar between groups, patients with punding showed a narrower dose range than those without punding. However, the low prevalence of punding limits the interpretation of this finding. The duration of treatment did not correlate with BIS-11 or any ICD. The literature suggests that the development of ICDs is not dose-dependent, and that even relatively low doses of DA can be associated with these behaviors [9, 13, 22, 23].

Finally, the strengths of our study include a relatively large sample size of patients with prolactinoma and the use of a healthy control group with characteristics similar to those of the study population. Some limitations should be acknowledged. The cross-sectional design and the use of ICD assessment tools not validated in prolactinoma populations may limit the interpretation of our findings. To date, no instruments have been specifically validated for the assessment of ICDs in patients with prolactinoma. Therefore, we used questionnaires previously employed in the literature, which contribute to the currently available evidence in this field. As impulsivity, ICDs, and psychiatric history were assessed through self-report measures rather than formal psychiatric interviews, misclassification cannot be excluded. However, the same questionnaires were applied to both patients and controls, reducing the risk that the differences observed between groups were driven by the assessment method itself. Furthermore, formal psychiatric evaluation of all patients treated with DA may not be feasible in routine clinical practice. In tertiary referral centers, psychiatric services are often dedicated to the management of patients with complex mental health disorders and may have limited capacity to evaluate all endocrine patients for screening purposes. In this context, screening instruments may play an important role in identifying individuals at higher risk of impulsivity or ICDs who would benefit from more comprehensive psychiatric assessment. Future studies are needed to evaluate the psychometric properties and screening performance of these instruments in patients with prolactinoma. In addition, psychiatric comorbidities were not formally assessed. Although information regarding self-reported anxiety, depression, and psychiatric medication was collected, no validated instruments were used to establish these diagnoses, and therefore their frequencies may have been under- or over-estimated. Furthermore, other psychiatric conditions associated with impulsivity were not systematically evaluated and may have contributed to residual confounding. Hormonal status was also not systematically assessed. Because a substantial proportion of participants were recruited through online questionnaires, detailed clinical and biochemical data were not available for all patients. In addition, the low frequency of specific ICDs may have limited the statistical power for subgroup analyses, despite the relatively large sample size. Lastly, part of the sample was recruited through online questionnaires, which may have introduced selection bias.

In conclusion, patients with prolactinoma treated with cabergoline exhibit increased impulsivity, particularly in the attentional domain, along with higher odds of compulsive shopping compared with healthy controls. These findings support a role for dopaminergic modulation in impulsivity. Importantly, lower educational level emerged as a consistent factor associated with both impulsivity and compulsive shopping, suggesting that sociodemographic factors may play a significant role in shaping vulnerability to these behaviors, in addition to the effects of dopaminergic treatment. These results highlight the importance of considering clinical and sociodemographic factors in the assessment of impulsivity and ICDs in patients with prolactinoma and may have implications for risk stratification and patient counseling in clinical practice. They also emphasize the importance of actively assessing impulsivity and impulse-related behaviors during clinical follow-up. Although not specifically validated for patients with prolactinoma, standardized instruments such as the BIS-11 and ICD-specific questionnaires may represent useful screening tools for identifying patients who could benefit from further psychiatric evaluation. Longitudinal studies are warranted to evaluate behavioral changes within individuals from before the initiation of dopamine agonist therapy through long-term treatment.

## Supplementary Information

Below is the link to the electronic supplementary material.

Supplementary Material 1

## Data Availability

No datasets were generated or analysed during the current study.
