# Supplementary material for: Increased impulsivity and higher odds of compulsive shopping among cabergoline-treated patients with prolactinoma: a case-control study
Source: Pituitary. 2026 Jul 24;29(4):130. doi: 10.1007/s11102-026-01728-z (PMC13400692; doi:10.1007/s11102-026-01728-z)

Table S1. BIS-11 total score

| Characteristic*^1^* | Univariable Analysis | | | Multivariable Analysis | | |
| --- | --- | --- | --- | --- | --- | --- |
|  | exp(Beta)*^1^* | 95% CI*^1^* | p-value*^1^* | exp(Beta)*^1^* | 95% CI*^1^* | p-value*^1^* |
| Group |  |  | 0.039 |  |  | 0.015 |
| Control | — | — |  | — | — |  |
| Patient | 1.05 | 1.00, 1.09 |  | 1.08 | 1.01, 1.15 |  |
| Age | 1.00 | 1.00, 1.00 | 0.4 | 1.00 | 1.00, 1.00 | 0.2 |
| Sex |  |  | 0.019 |  |  | 0.10 |
| F | — | — |  | — | — |  |
| M | 0.95 | 0.90, 0.99 |  | 0.96 | 0.92, 1.01 |  |
| Education |  |  | <0.001 |  |  | <0.001 |
| College degree | — | — |  | — | — |  |
| High school or less | 1.13 | 1.08, 1.17 |  | 1.16 | 1.09, 1.23 |  |
| Group * Education |  |  |  |  |  | 0.083 |
| Patient * High school or less |  |  |  | 0.93 | 0.86, 1.01 |  |
| *^1^* Final Model (Gamma (Log)) \| AIC: 1954.85 \| Explained Deviance: 14.59% \| *Estimates exponentiated (Ratio of Means). | | | | | | |
| Abbreviation: CI = Confidence Interval | | | | | | |

Table S2. BIS-11 motor impulsivity

| Characteristic*^1^* | Univariable Analysis | | | Multivariable Analysis | | |
| --- | --- | --- | --- | --- | --- | --- |
|  | exp(Beta)*^1^* | 95% CI*^1^* | p-value*^1^* | exp(Beta)*^1^* | 95% CI*^1^* | p-value*^1^* |
| Group |  |  | 0.4 |  |  | 0.070 |
| Control | — | — |  | — | — |  |
| Patient | 1.03 | 0.97, 1.08 |  | 1.08 | 0.99, 1.16 |  |
| Age | 1.00 | 1.00, 1.00 | 0.5 | 1.00 | 1.00, 1.00 | 0.4 |
| Sex |  |  | 0.2 |  |  | 0.4 |
| F | — | — |  | — | — |  |
| M | 0.96 | 0.91, 1.02 |  | 0.98 | 0.92, 1.04 |  |
| Education |  |  | <0.001 |  |  | <0.001 |
| College degree | — | — |  | — | — |  |
| High school or less | 1.10 | 1.04, 1.16 |  | 1.15 | 1.07, 1.24 |  |
| Group * Education |  |  |  |  |  | 0.066 |
| Patient * High school or less |  |  |  | 0.90 | 0.81, 1.01 |  |
| *^1^* Final Model (Gamma (Log)) \| AIC: 1474.52 \| Explained Deviance: 6.43% \| *Estimates exponentiated (Ratio of Means). | | | | | | |
| Abbreviation: CI = Confidence Interval | | | | | | |
|  | | | | | | |

Table S3. BIS-11 non-planning impulsivity

| Characteristic*^1^* | Univariable Analysis | | | Multivariable Analysis | | |
| --- | --- | --- | --- | --- | --- | --- |
|  | exp(Beta)*^1^* | 95% CI*^1^* | p-value*^1^* | exp(Beta)*^1^* | 95% CI*^1^* | p-value*^1^* |
| Group |  |  | 0.021 |  |  | 0.087 |
| Control | — | — |  | — | — |  |
| Patient | 1.06 | 1.01, 1.11 |  | 1.06 | 0.99, 1.14 |  |
| Age | 1.00 | 1.00, 1.00 | >0.9 | 1.00 | 1.00, 1.00 | 0.6 |
| Sex |  |  | 0.038 |  |  | 0.082 |
| F | — | — |  | — | — |  |
| M | 0.95 | 0.90, 1.00 |  | 0.96 | 0.91, 1.01 |  |
| Education |  |  | <0.001 |  |  | <0.001 |
| College degree | — | — |  | — | — |  |
| High school or less | 1.17 | 1.12, 1.22 |  | 1.18 | 1.11, 1.26 |  |
| Group * Education |  |  |  |  |  | 0.4 |
| Patient * High school or less |  |  |  | 0.96 | 0.88, 1.05 |  |
| *^1^* Final Model (Gaussian (Log)) \| AIC: 1547.8 \| Explained Deviance: 17.5% \| *Estimates exponentiated (Ratio of Means). | | | | | | |
| Abbreviation: CI = Confidence Interval | | | | | | |

Table S4. BIS-11 attentional impulsivity

| Characteristic*^1^* | Univariable Analysis | | | Multivariable Analysis | | |
| --- | --- | --- | --- | --- | --- | --- |
|  | exp(Beta)*^1^* | 95% CI*^1^* | p-value*^1^* | exp(Beta)*^1^* | 95% CI*^1^* | p-value*^1^* |
| Group |  |  | 0.090 |  |  | 0.025 |
| Control | — | — |  | — | — |  |
| Patient | 1.06 | 0.99, 1.13 |  | 1.11 | 1.01, 1.22 |  |
| Age | 1.00 | 1.00, 1.00 | 0.11 | 1.00 | 1.0, 1.00 | 0.078 |
| Sex |  |  | 0.025 |  |  | 0.089 |
| F | — | — |  | — | — |  |
| M | 0.93 | 0.86, 0.99 |  | 0.94 | 0.88, 1.01 |  |
| Education |  |  | 0.004 |  |  | 0.003 |
| College degree | — | — |  | — | — |  |
| High school or less | 1.10 | 1.03, 1.17 |  | 1.14 | 1.05, 1.25 |  |
| Group * Education |  |  |  |  |  | 0.11 |
| Patient * High school or less |  |  |  | 0.90 | 0.80, 1.02 |  |
| *^1^* Final Model (Gamma (Log)) \| AIC: 1474.33 \| Explained Deviance: 7.44% \| *Estimates exponentiated (Ratio of Means). | | | | | | |
| Abbreviation: CI = Confidence Interval | | | | | | |

Table S5. BIS-11 score ≥60.

| Characteristic*^1^* | Univariable Analysis | | | Multivariable Analysis | | |
| --- | --- | --- | --- | --- | --- | --- |
|  | OR*^1^* | 95% CI*^1^* | p-value*^1^* | OR*^1^* | 95% CI*^1^* | p-value*^1^* |
| Group |  |  | 0.001 |  |  | 0.015 |
| Control | — | — |  | — | — |  |
| Patient | 2.24 | 1.37, 3.68 |  | 2.58 | 1.20, 5.70 |  |
| Age | 0.99 | 0.97, 1.01 | 0.5 | 0.99 | 0.97, 1.01 | 0.2 |
| Sex |  |  | 0.2 |  |  | 0.4 |
| F | — | — |  | — | — |  |
| M | 0.72 | 0.43, 1.21 |  | 0.80 | 0.46, 1.40 |  |
| Education |  |  | <0.001 |  |  | <0.001 |
| College degree | — | — |  | — | — |  |
| High school or less | 3.34 | 2.02, 5.59 |  | 3.86 | 1.85, 8.35 |  |
| Group * Education |  |  |  |  |  | 0.5 |
| Patient * High school or less |  |  |  | 0.70 | 0.25, 1.98 |  |
| *^1^* Final Model (Logistic) \| AIC: 342.17 \| Explained Deviance (Pseudo-R2): 9.09% \| *Estimates exponentiated (Odds Ratio). | | | | | | |
| Abbreviations: CI = Confidence Interval, OR = Odds Ratio | | | | | | |

Table S6. Punding

| Characteristic*^1^* | Univariable Analysis | | | Multivariable Analysis | | |
| --- | --- | --- | --- | --- | --- | --- |
|  | OR*^1^* | 95% CI*^1^* | p-value*^1^* | OR*^1^* | 95% CI*^1^* | p-value*^1^* |
| Group |  |  | 0.6 |  |  | 0.3 |
| Control | — | — |  | — | — |  |
| Patient | 0.79 | 0.29, 2.06 |  | 2.17 | 0.53, 9.45 |  |
| Age | 0.93 | 0.87, 0.97 | 0.002 | 0.93 | 0.87, 0.97 | 0.002 |
| Sex |  |  | 0.7 |  |  | >0.9 |
| F | — | — |  | — | — |  |
| M | 0.80 | 0.25, 2.21 |  | 0.94 | 0.28, 2.73 |  |
| Education |  |  | 0.7 |  |  | 0.4 |
| College degree | — | — |  | — | — |  |
| High school or less | 0.85 | 0.32, 2.24 |  | 1.85 | 0.48, 7.81 |  |
| Group * Education |  |  |  |  |  | 0.095 |
| Patient * High school or less |  |  |  | 0.18 | 0.02, 1.34 |  |
| *^1^* Final Model (Logistic) \| AIC: 130.64 \| Explained Deviance (Pseudo-R2): 9.54% \| *Estimates exponentiated (Odds Ratio). | | | | | | |
| Abbreviations: CI = Confidence Interval, OR = Odds Ratio | | | | | | |

Table S7. Compulsive shopping

| Characteristic*^1^* | Univariable Analysis | | | Multivariable Analysis | | |
| --- | --- | --- | --- | --- | --- | --- |
|  | OR*^1^* | 95% CI*^1^* | p-value*^1^* | OR*^1^* | 95% CI*^1^* | p-value*^1^* |
| Group |  |  | >0.9 |  |  | 0.015 |
| Control | — | — |  | — | — |  |
| Patient | 1.00 | 0.49, 2.03 |  | 4.47 | 1.33, 17.8 |  |
| Age | 0.97 | 0.94, 1.00 | 0.078 | 0.97 | 0.94, 1.00 | 0.093 |
| Sex |  |  | <0.001 |  |  | <0.001 |
| F | — | — |  | — | — |  |
| M | 0.10 | 0.02, 0.35 |  | 0.11 | 0.02, 0.38 |  |
| Education |  |  | 0.3 |  |  | 0.008 |
| College degree | — | — |  | — | — |  |
| High school or less | 1.41 | 0.69, 2.96 |  | 4.52 | 1.46, 17.2 |  |
| Group * Education |  |  |  |  |  | 0.002 |
| Patient * High school or less |  |  |  | 0.09 | 0.02, 0.43 |  |
| *^1^* Final Model (Logistic) \| AIC: 192.49 \| Explained Deviance (Pseudo-R2): 13.94% \| *Estimates exponentiated (Odds Ratio). | | | | | | |
| Abbreviations: CI = Confidence Interval, OR = Odds Ratio | | | | | | |

Table S8. Predicted probabilities of compulsive shopping according to study group and educational level

| Estimated Marginal Means (Probabilities) | | | |
| --- | --- | --- | --- |
| Scenario (Group * Education) | Predicted Probability | Lower 95% CI | Upper 95% CI |
| Control - College degree | 0.027 | 0.008 | 0.086 |
| Patient - College degree | 0.111 | 0.047 | 0.239 |
| Control - High school or less | 0.112 | 0.048 | 0.238 |
| Patient - High school or less | 0.049 | 0.019 | 0.122 |
| Estimates represent predicted probabilities on a 0 to 1 scale. Model: Logistic | | | |

Table S9. Hypersexuality

| Characteristic*^1^* | Univariable Analysis | | | Multivariable Analysis | | |
| --- | --- | --- | --- | --- | --- | --- |
|  | OR*^1^* | 95% CI*^1^* | p-value*^1^* | OR*^1^* | 95% CI*^1^* | p-value*^1^* |
| Group |  |  | 0.12 |  |  | 0.2 |
| Control | — | — |  | — | — |  |
| Patient | 2.78 | 0.78, 12.9 |  | 2.92 | 0.53, 22.2 |  |
| Age | 0.98 | 0.93, 1.03 | 0.5 | 0.98 | 0.92, 1.03 | 0.4 |
| Sex |  |  | 0.028 |  |  | 0.029 |
| F | — | — |  | — | — |  |
| M | 3.95 | 1.16, 15.5 |  | 4.00 | 1.15, 15.9 |  |
| Education |  |  | 0.6 |  |  | 0.8 |
| College degree | — | — |  | — | — |  |
| High school or less | 0.70 | 0.20, 2.40 |  | 0.69 | 0.03, 7.63 |  |
| Group * Education |  |  |  |  |  | >0.9 |
| Patient * High school or less |  |  |  | 1.06 | 0.06, 30.3 |  |
| *^1^* Final Model (Logistic) \| AIC: 94.85 \| Explained Deviance (Pseudo-R2): 9.24% \| *Estimates exponentiated (Odds Ratio). | | | | | | |
| Abbreviations: CI = Confidence Interval, OR = Odds Ratio | | | | | | |
|  | | | | | | |

Table S10. Gambling

| Characteristic*^1^* | Univariable Analysis | | | Multivariable Analysis | | |
| --- | --- | --- | --- | --- | --- | --- |
|  | OR*^1^* | 95% CI*^1^* | p-value*^1^* | OR*^1^* | 95% CI*^1^* | p-value*^1^* |
| Group |  |  | 0.7 |  |  | 0.8 |
| Control | — | — |  | — | — |  |
| Patient | 1.26 | 0.33, 5.19 |  | 1.38 | 0.05, 36.2 |  |
| Age | 0.98 | 0.92, 1.03 | 0.4 | 0.96 | 0.90, 1.02 | 0.2 |
| Sex |  |  | 0.030 |  |  | 0.013 |
| F | — | — |  | — | — |  |
| M | 4.49 | 1.15, 21.7 |  | 5.90 | 1.44, 30.1 |  |
| Education |  |  | 0.13 |  |  | 0.14 |
| College degree | — | — |  | — | — |  |
| High school or less | 3.11 | 0.73, 21.1 |  | 5.04 | 0.59, 107 |  |
| Group * Education |  |  |  |  |  | 0.8 |
| Patient * High school or less |  |  |  | 0.67 | 0.02, 23.8 |  |
| *^1^* Final Model (Logistic) \| AIC: 81.15 \| Explained Deviance (Pseudo-R2): 11.76% \| *Estimates exponentiated (Odds Ratio). | | | | | | |
| Abbreviations: CI = Confidence Interval, OR = Odds Ratio | | | | | | |
|  | | | | | | |

Supplementary Figure S1. Distribution of total BIS-11 scores among controls and patients treated with cabergoline


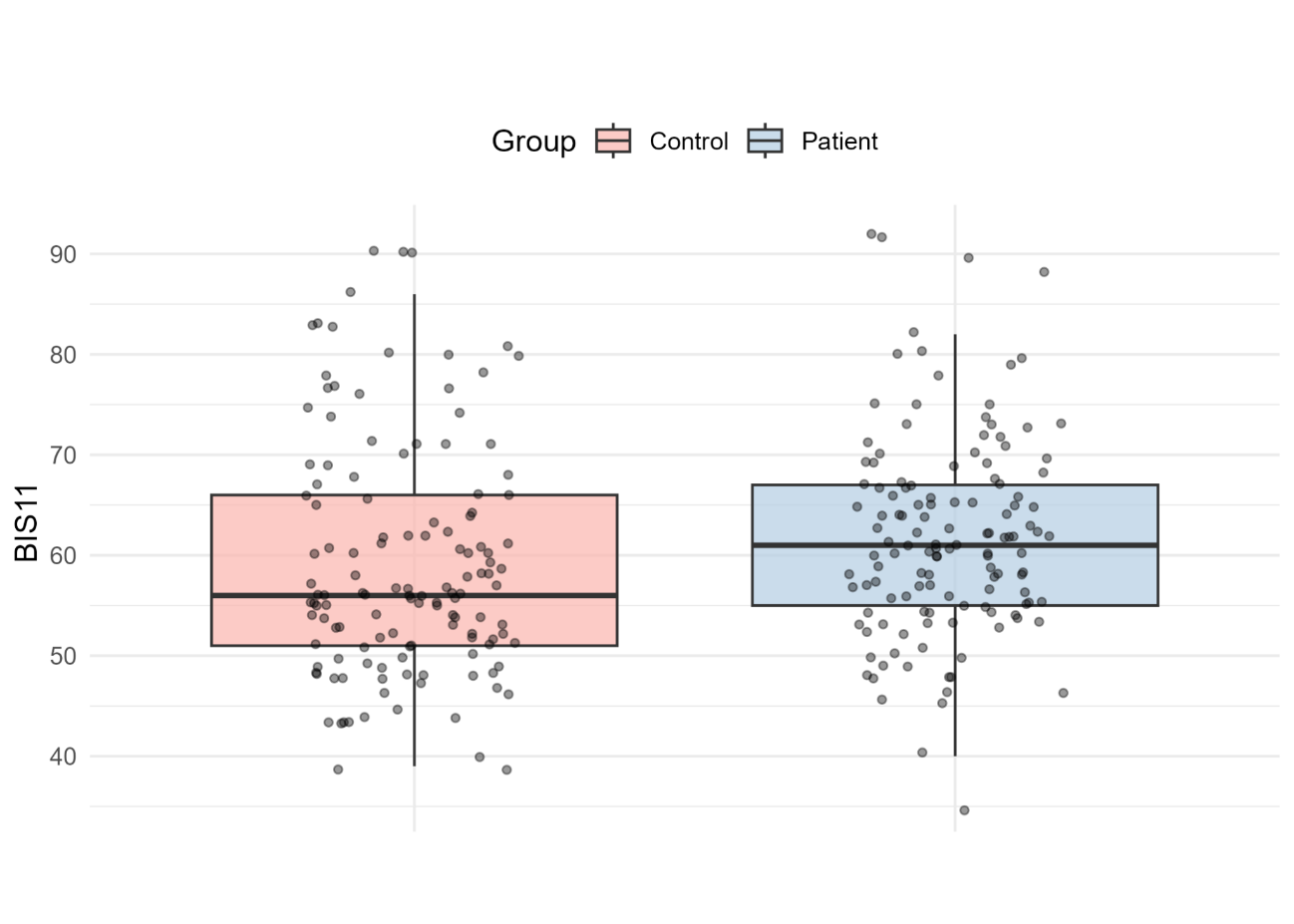

Supplement: Supplementary file 1 — Supplementary Material 1 [file 11102_2026_1728_MOESM1_ESM.docx]
